# Engineering the Oleaginous Yeast *Rhodosporidium toruloides* for Improved Resistance Against Inhibitors in Biomass Hydrolysates

**DOI:** 10.3389/fbioe.2021.768934

**Published:** 2021-11-15

**Authors:** Liting Lyu, Yadong Chu, Sufang Zhang, Yue Zhang, Qitian Huang, Shuang Wang, Zongbao K. Zhao

**Affiliations:** ^1^ Laboratory of Biotechnology, Dalian Institute of Chemical Physics (CAS), Dalian, China; ^2^ Dalian Key Laboratory of Energy Biotechnology, Dalian Institute of Chemical Physics (CAS), Dalian, China; ^3^ State Key Laboratory of Catalysis, Dalian Institute of Chemical Physics, Chinese Academy of Sciences, Dalian, China

**Keywords:** *Rhodosporidium toruloides*, microbial lipids, lignocellulose, inhibitors, genetic engineering, screening

## Abstract

Conversion of lignocellulosic biomass into lipids and related chemicals has attracted much attention in the past two decades, and the oleaginous yeast *Rhodosporidium* *toruloides* has been widely used in this area. While *R. toruloides* species naturally have physiological advantages in terms of substrate utilization, lipid accumulation, and inhibitor resistance, reduced lipid production and cell growth are noticed when biomass hydrolysates are used as feedstocks. To improve the robustness of *R. toruloides*, here, we devised engineered strains by overexpressing genes responsible for phenolic compound degradation. Specifically, gene expression cassettes of the manganese peroxidase gene (MNP) and versatile peroxidase gene (VP) were constructed and integrated into the genome of *R. toruloides* NP11. A series of engineered strains were evaluated for lipid production in the presence of typical phenolic inhibitors. The results showed that *R. toruloides* strains with proper expression of MNP or VP indeed grew faster in the presence of vanillin and 5-hydroxymethylfurfural than the parental strain. When cultivated in concentrated mode biomass hydrolysates, the strain VP18 had improved performance as the cell mass and lipid content increased by 30% and 25%, respectively. This study provides more robust oleaginous yeast strains for microbial lipid production from lignocellulosic biomass, and similar efforts may be used to devise more advanced lipid producers.

## Introduction

The bioconversion of lignocellulose into biofuels and other high value-added chemicals is of significant potential for its environmental protection and energy sustainability (Jin et al., 2015). Lignocellulose contains roughly 40% of cellulose, 25% of hemicellulose, and 20% of lignin (Kim et al., 2021). After pretreatment and hydrolysis, cellulose and hemicellulose are hydrolyzed into sugars, along with the formation of some inhibitory compounds, including acids (formic, acetic, and levulinic acids) and furnaldehydes (furfural and 5-hydroxymethylfurfural HMF) (Tramontina et al., 2020). Lignin is a phenolic heteropolymer composed of p-hydroxyphenyl, guaiacyl, and syringyl groups (Chen and Wan, 2017). During chemical or biological pretreatment, lignin is degraded into monomeric compounds, mainly as p-hydroxybenzaldehyde (PHB), vanillin, syringaldehyde, and their corresponding reduced or oxidized products (Zhu et al., 2017; Osorio–González et al., 2019). These lignin-derived phenols, associated with organic acids and furnaldehydes, usually have inhibitive and toxic effects on microorganisms, thus playing negative roles in biological processes (Ragauskas et al., 2014).

*Rhodosporidium toruloides* is a typical oleaginous yeast. It is considered an ideal platform for the bioconversion of lignocellulose into fine chemical products (Wen et al., 2020). This yeast can generate cell mass up to a high density of 130 g/L with a capacity to produce lipids over 70% of its dry cell weight (Li et al., 2007; Park et al., 2018). Besides being endowed with an endogenous mevalonate pathway for the synthesis of carotenoids and some other terpenes (Jiao et al., 2019; Zhuang et al., 2019; Geiselman et al., 2020), *R. toruloides* is also designed with the ability to utilize a number of biomass and carbon sources. As a robust organism, this yeast has demonstrated to utilize some forms of biomass-derived inhibitors (Hu and Zhao, 2009; Tramontina et al., 2020). However, study has shown that *R. toruloides* are strongly inhibited by 1 g/L of furfuran, PHB, or vanillin (Hu and Zhao, 2009; Zhao et al., 2012). This therefore suggests a weaker tolerance and the utilization ability of *R. toruloides* to phenolic aldehydes and furnaldehydes. Specially, in order to improve the product yield, the hydrolysate is usually concentrated to increase the sugar content, resulting in a corresponding rise of by-products (Mussatto, 2004; Agu et al., 2016). Thus, it is challenging for *R. toruloides* to improve robustness during fermentation.

Fungal lignin peroxidase (Lip), manganese peroxidase (Mnp), and versatile peroxidase (Vp), usually participating in lignin degradation, have shown relevance in the detoxification of lignocellulosic hydrolysates (Tramontina et al., 2020). Lip catalyzes H_2_O_2_-dependent oxidative depolymerization, resulting in demethylation and intramolecular rearrangements of lignin-derived phenols (Chandra et al., 2017). Mnp is also a known phenol-oxidizing enzyme that decomposes the substrate by forming a radical on the phenolic hydroxyl group. In addition, Mnp is also reported to degrade non-phenolic compounds by generating a peroxyl radical (Watanabe et al., 2000). A peroxyl radical can degrade bonds in a number of different structures, including a non-phenolic β-O-4–linked lignin, polyethylene, and phenanthrene. Due to their strong oxidizing properties, Mnp has recently been reported to degrade furfural and HMF (Yee et al., 2018). Vp is a hybrid peroxidase that has catalytic functions of both Lip and Mnp (Tramontina et al., 2020).

**FIGURE 1:**
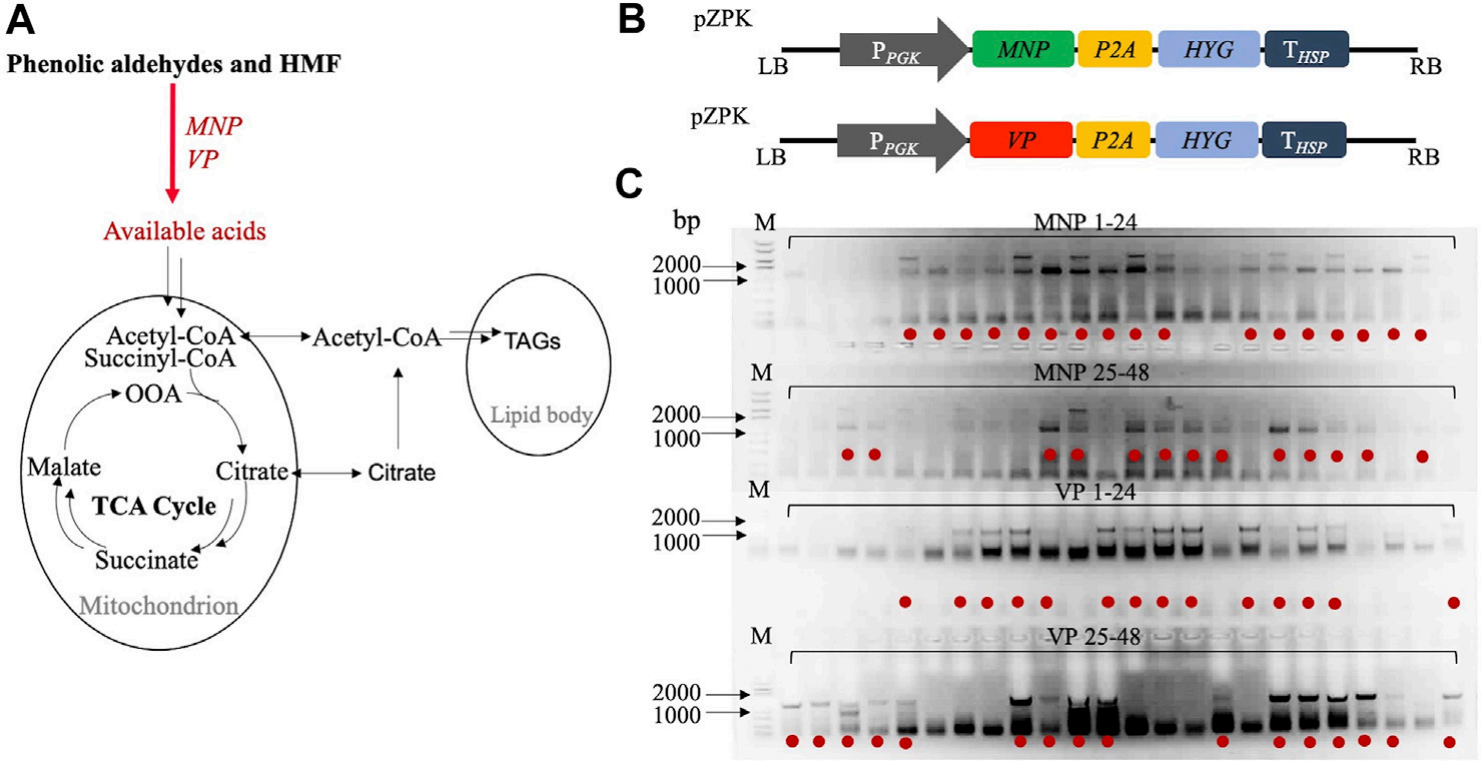
Integration of exogenous genes *MNP* and *VP* with *R. toruloides* chromosomes: **(A)** hypothetic metabolize pathway of phenolic aldehydes and HMF; **(B)** information of gene expression cassettes; **(C)** *R. toruloides* colony PCR results for *MNP* and *VP* genes verification. Red dots represent right genotypes chose for further screening.

Here, we hypothesize that engineered *R. toruloides* could improve the robustness of *R. toruloides*. This strategy includes the overexpressing the manganese peroxidase gene (*MNP*) and the versatile peroxidase gene (*VP*) responsible for by-products degradation (Figure 1A). After high-throughput screening, fermentation was employed to investigate the lipid production and typical inhibitor utilization of the engineered strains. This study provides more robust oleaginous yeast strains for microbial lipid production from lignocellulosic biomass, and similar efforts may be used to devise more advanced lipid producers.

## Materials and Methods

### Strains, Media, and Reagents

All strains used in this study are listed in Supplementary Table S1. *Rhodosporidium toruloides* CGMCC 2.1389 was purchased from the China General Microbiological Culture Collection Centre (Beijing, China), and *Rhodosporidium toruloides* NP11 was its haploid separated by our laboratory (Zhu et al., 2012). *Escherichia coli* DH10B and *Agrobacterium tumefaciens* AGL1 used for routine molecular manipulation were cultured in the Luria–Bertani medium (10.0 g/L NaCl, 10.0 g/L tryptone, and 5.0 g/L yeast extract) at 37°C and 30°C, respectively. Yeast cells were cultured in the yeast extract–peptone–dextrose (YPD) medium (20.0 g/L glucose, 10.0 g/L yeast extract, and 20.0 g/L peptone) at 30°C. Antibiotics were used at the following concentrations: kanamycin of 50 μg/ml and hygromycin of 50 μg/ml.

The media used in preliminary screening consist of yeast nitrogen base (YNB), with 2.5 g/L of PHB, vanillin, syringaldehyde or HMF as the sole carbon source, 1.0 g/L of KH_2_PO_4_. Mother selection media used for secondary screening were as follows: PHB (2.5 g/L), vanillin (2.5 g/L), syringaldehyde (2.5 g/L), and HMF (5.0 g/L) which served as the sole carbon source in the YNB medium. Each of these media, respectively, contains 1.0 g/L of KH_2_PO_4_.

The concentrated model hydrolysate was made up of by-products such as vanillin 16 g/L, PHB 10 g/L, syringaldehyde 15 g/L, vanillic acid 4 g/L, p-hydroxybenzoic acid 4 g/L, vanillyl alcohol 4 g/L, syringic acid 5 g/L, guaiacol 1 g/L, syringol 2 g/L, and HMF 5 g/L. These by-products were dissolved in ddH_2_O and filter-sterilized. The nitrogen-limited medium was formulated with glucose 70.0 g/L, yeast extract 0.8 g/L, (NH_4_)_2_SO_4_ 0.1 g/L, MgSO_4_ 1.5 g/L, and KH_2_SO_4_ 1.0 g/L at a C/N ratio of 100 and then sterilized using an autoclave. As a fermentation substrate, a concentrated model hydrolysate was ten times diluted in the nitrogen-limited medium. The initial pH of the substrate was adjusted to 5.6 using 3 M of NaOH.

The aforementioned by-products and chemicals were purchased from Aladdin (Shanghai, China). Yeast extract, peptone, and YNB were from Oxoid (Basingstoke, Hampshire, United Kingdom). Enzymes used for PCR amplification were purchased from Takara (Dalian, China). DNA gel purification and plasmid extraction kits were supplied by Sangon Biotech (Shanghai, China). All other chemicals were purchased from Bonuo Biological and Chemical Reagent Co. (Dalian, China).

### Plasmid Construction, Transformation, and Verification

To construct plasmids pZPK-*MNP-HYG* and pZPK-*VP-HYG*, *MNP* (GenBank: M60672.1) gene from *Phanerochaete chrysosporium* and *VP* (GenBank: AF175710.1) gene from *Pleurotus eryngii* were codon-optimized according to the codon preference of *R. toruloides*, synthesized by Synbio Technologies (Suzhou, China) and linked to the pUC57 plasmid. First, the primer pair *P2A*-*HYG*-F and *HYG*-T_*HSP*_-R was used to amplify the fragment *P2A*-*HYG*-T_*HSP*_ from the plasmid pJX14 (Jiao et al., 2018). The fragments were then assembled into the plasmid pJX14 to obtain pZPK-P_*PGK*_ *-HYG-P2A-HYG-*T_*HSP*_ by the RF cloning method (Van den Ent and Löwe, 2006). Primer pairs P_PGK_-*MNP/VP*-F and *MNP/VP-P2A*-R were used, respectively, to amplify P_PGK_ *-MNP*-*P2A* and P_PGK_ *-VP-P2A* fragments from pUC57 plasmids. The fragments were then assembled into the plasmid pZPK-P_*PGK*_ *-HYG-P2A-HYG-*T_*HSP*_ by the RF cloning method. The genotype of the clone was verified by Sanger sequencing (Synbio Technologies, Suzhou, China). All plasmids and primers used in this study are listed in Supplementary Tables S1, S2, respectively.

The correct plasmids pZPK-*MNP-HYG* and pZPK-*VP-HYG* were transformed into AGL1 by electroporation, and strains were selected on LB plates supplemented with 50 μg/ml kanamycin. The transformation of the *R. toruloides* was done according to a published *Agrobacterium tumefaciens*–mediated transformation (ATMT) method (Lin et al., 2014). *R. toruloides* colony PCR analysis for transformant verification was assayed according to previous reports (Lin et al., 2012; Lin et al., 2014). Primer pairs used in this study were P_*PGK*_-*MNP*-F/*HYG*-R and P_*PGK*_-*VP*-F/*HYG*-R, as shown in Table S2.

### Preliminary and Secondary Screening

Randomized selected transformants from selective plates were subjected to repeated sub-cultivation 5 times. A single colony of the transformant was added to 3 ml of YPD medium in a 24-well plate for 24-h culturing to prepare seeds.

For preliminary screening, 15 µL of the seed broth was added to 400 µL of selection medium in 96-well plates (Corning 3960, Corning, NY). For secondary screening, the seed broth was inoculated into the selection medium in 96-well plates to make an initial optical density (measured at a wavelength of 600 nm, OD_600_) of 0.2. Mother media were diluted into four concentration gradients to keep phenolic aldehydes to 2.5-, 1.2-, 0.6-, and 0.3 g/L, and HMF to 5.0-, 2.5-, 1.3- and 0.6 g/L, respectively. All plates were covered with a sterile breathable sealing film (Axygen, United States), vortexed in a Multitron (ZQZY-88BH, Shanghai Zhichu Instrument Co., Ltd., China) at 30°C and 800 rpm for 48 h. The samples were collected after 48 h to measure OD_600_. Inoculation and measurement processes were done by the fluid handing workstation (Biomek i7) combined with an automation workflow scheduling software (SAMI EX) and a microplate reader and controlled by Beckman software (Beckman Coulter Inc., United States) (Sobreira et al., 2020).

### Fermentation and Lipid Production Procedures

Seed cultures were prepared by inoculating a single colony from selective plates into 50 ml YPD medium for 24 h; 5 ml of seed cultures were added to 45 ml nitrogen-limited medium in a 250-ml shake flask at 30°C and 200 rpm for 152 h. Fermentation experiments were performed in triplicate.

During fermentation in the nitrogen-limited substrate, glucose concentration and OD_600_ were quantified every 24 h by a glucose analyzer (SBA-50B; Shandong Academy of Sciences, Jinan, China) and a spectrophotometer (Evolution 220; Thermo, United States). At the fermentation endpoint, the lipids were extracted and detected, as described previously (Zhu et al., 2015). By-product degradation was analyzed by thin layer chromatography (TLC) with chloroform and ethyl acetate (3:1, v/v) as the elution solvent and was visualized under iodine vapor and UV 230 nm.

## Results

### Engineering *R. toruloides* and Verification

Plasmids were constructed using the RF cloning method (Figure 1B) and transformed into *R. toruloides* using the ATMT method, which resulted in the random chromosomal integration of transgenes. To further ensure the integration of exogenous genes, the colony PCR analysis was carried out (Figure 1C). The results clearly indicated the presence of *MNP* and *VP* genes for most transformants. For each genotype, thirty of the right transformants were chosen for the following experiment.

### Screening of Engineered Strains

Thirty transformants of each genotype were selected for preliminary screening. The selection medium was prepared by the YNB substrate with four different kinds of inhibitors as the sole carbon source. These inhibitors, “phenolic aldehydes, PHB, vanillin, and syringaldehyde,” were selected to represent the typical lignin degradation monomers of hydroxyphenyl, guaiacyl, and syringyl groups. All the transformants were cultured for 48 h, and the initial and the terminal OD_600_ were detected using the Biomek i7 fluid handling workstation. The results showed that 2.5 g/L of PHB, vanillin, and syringaldehyde, except HMF, inhibited the cell growth of all strains (Supplementary Figures S1, S2). Thus, seven strains with the highest cell density under 2.5 g/L of HMF were selected as dominant strains.

**FIGURE 2:**
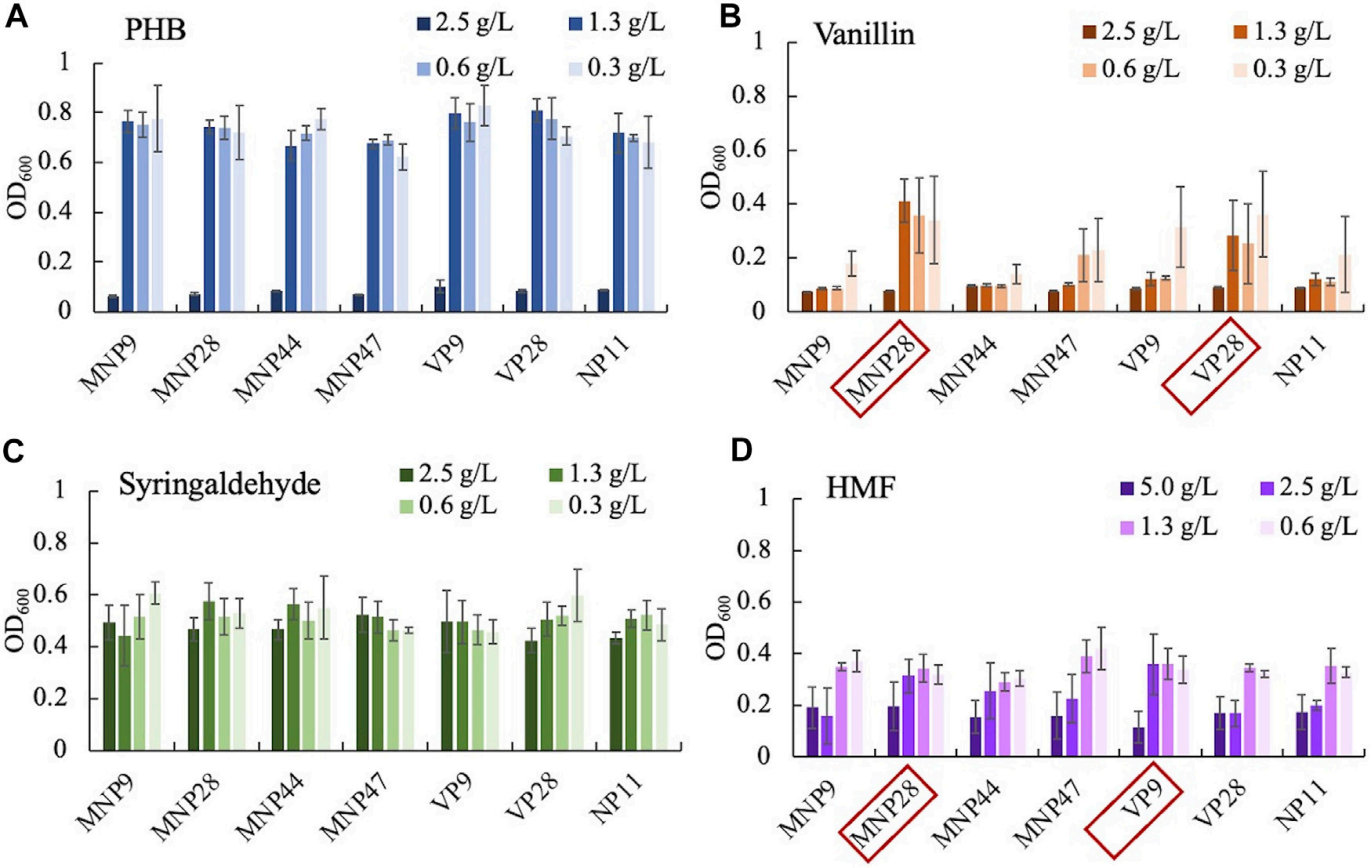
Growth of dominant strains and the control under gradient concentrations (g/L) of PHB-p-hydroxy benzaldehyde **(A)**, vanillin **(B)**, syringaldehyde **(C),** and HMF-hydroxymethyl furfural **(D)**. Initial OD_600_ was controlled at 0.2 by computer programming, and final OD_600_ was reached in 48 h. Red boxes represent the best growing strains selected for further test. Experiments were performed in triplicate.

For secondary screening, substrates within various inhibitors were set for four concentration gradients (Figure 2). Seven dominant strains and the control were cultured with an initial OD_600_ of 0.2 for 48 h and screened by detecting the highest OD_600_ under high concentrations. The strain MNP28 and VP28 showed an increase in cell growth under 1.3- and 0.6 g/L of vanillin compared with the control. The cell growth of strains MNP28 and VP9 had an increase under 2.5 g/L of HMF. These results suggest that some engineered strains could indeed utilize vanillin and HMF. All strains used in the study were found to grow under 1.3 g/L of PHB and 2.5 g/L of syringaldehyde, which indicated that the wild-type *R. toruloides* NP11 was naturally capable of using or tolerating certain concentrations of the two inhibitors. This finding is also consistent with our previous study (Hu and Zhao, 2009). In this study, concentrations of PHB and syringaldehyde concentrations were higher than those in real lignocellulose hydrolysates (Persson et al., 2002; Min et al., 2014). Thus, we did not explore the optimal concentration range of these two inhibitors. In addition, we selected the engineered strains (MNP28, VP9, and VP28) with superior performance for further test.

### Fermentation Performance of Engineered Strains

To detect the fermentation performance and the by-product utilization of engineered strains in a concentrated hydrolysate, a corn stover hydrolysate with three times the concentration was simulated. The composition of the model hydrolysate was carried out according to the alkaline pretreatment method (Persson et al., 2002; Mcintosh and Vancov, 2011; Min et al., 2014). First, it was necessary to test whether simulated hydrolysates inhibited *R. toruloides*. Two wild-type strains *R. toruloides* NP11 and CGMCC 2.1389 were fermented by the concentrated hydrolysate (Supplementary Figure S3). Compared with the control (wild strains cultured under the nitrogen-limited medium), the concentrated model hydrolysate showed serious inhibition on *R. toruloides* strains. After 48 h of fermentation, cell densities of the NP11 and the CGMCC 2.1389 were slightly decreased and with almost no glucose consumed.

**FIGURE 3:**
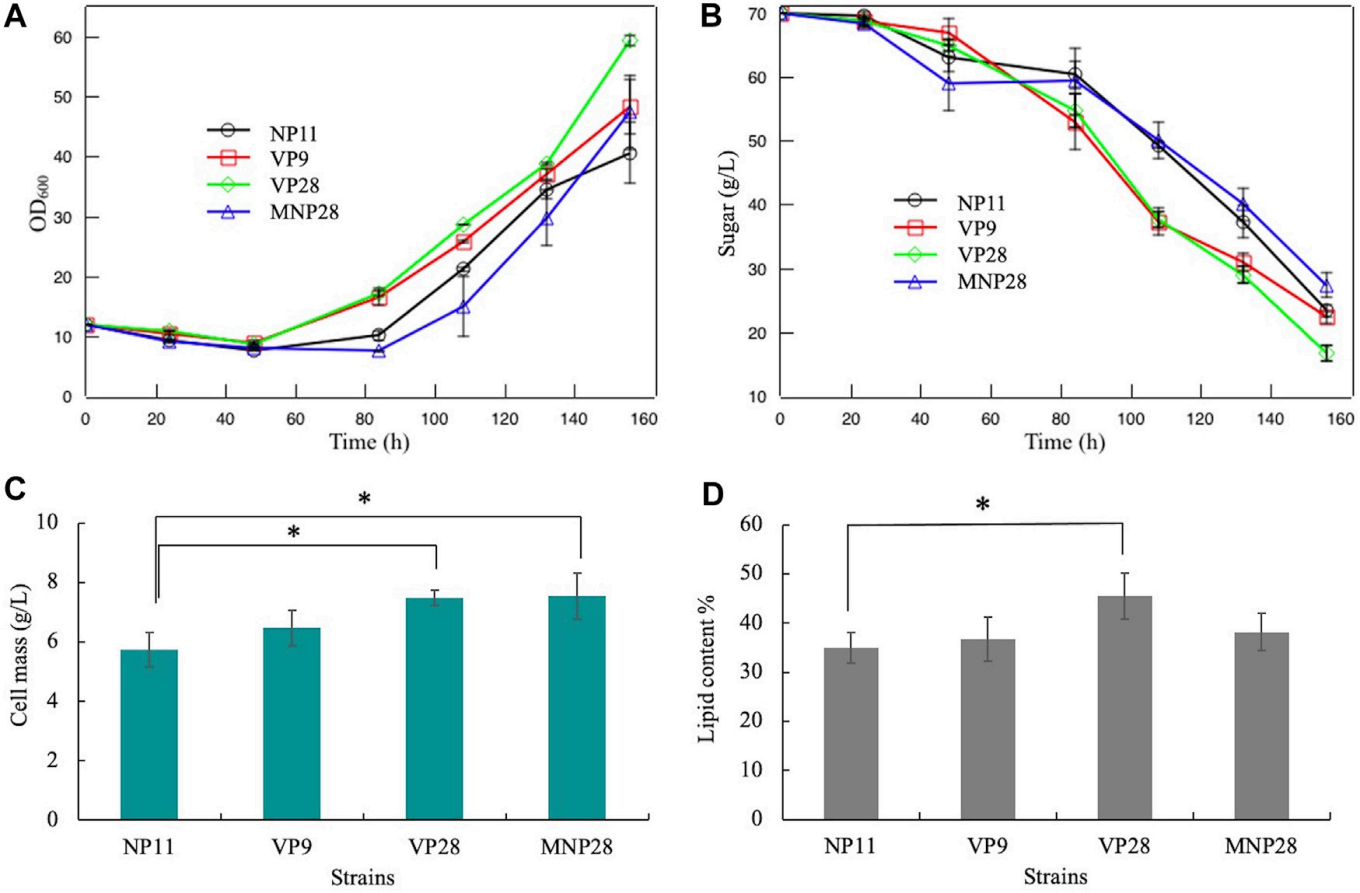
Fermentation performance of engineering strains and the control: **(A)** cell growth; **(B)** glucose consumption; **(C)** cell mass at the fermentation endpoint; **(D)** lipid content at the fermentation endpoint. Error bars represent the standard deviation of three independent experiments. Significantly different values were performed by the ANOVA and *post hoc* Dunnett test. * means *p* < 0.05.

Furthermore, the concentrated model hydrolysate was used as the fermentation substrate. As shown in Figures 3A,B, cell growth was still restrained in the first 48 h, and glucose was hardly consumed. Then strains VP9 and VP28 released form the by-product inhibition and had a quick recovery of cell growth and glucose consumption. These results suggest that the *VP* gene in *R. toruloides* play a more important role than the *MNP* during by-product degradation. Compared with the control, the VP28 had a significant improvement in cell mass and lipid accumulation, which increased by 30 and 25%, respectively (Figures 3C,D). This indicates that the *VP* gene had a positive effect on the bioconversion of lignin-derived phenols and HMF. The MNP28 strain also had an increase of 32% on cell mass but remained unchanged in the lipid content, which might be caused by its long inhibitory stage.

The by-product conversion of engineered strains was then examined by TLC (Supplementary Figure S4). Syringol and guaiacol could not be colored by iodine vapor due to their low concentrations in the simulated hydrolysate. The TLC result gave a qualitative analysis about lignin-derived phenol bioconversion. Three phenolic aldehydes and HMF were completely conversed within 48 h in all fermentation supernatants. The VP9 and the VP28 strains exhibited a faster bioconversion rate than the MNP47 and the control. p-Hydroxybenzyl alcohol generated along with the PHB consumption indicates that the endogenous aldehyde reductase of *R. toruloides* catalyzed the reduction of the PHB aldehyde group (Hu et al., 2018)*.* Compared with the control, three engineered strains rapidly convert p-hydroxybenzoic acid, vanillic acid, and syringic acid in 48 h without obvious accumulation (Supplementary Figure S5), thus suggesting an efficient decomposition of phenolic acids by Mnp and Vp enzymes.

## Discussion

This study integrated two peroxidase genes (*MNP* and *VP*) into the oleaginous yeast *R. toruloides*. After high-throughput screening, the strains MNP28, VP9, and VP28 with superior performance were chosen for fermentation. There were only three strains obtained from sixty transformants; this might be due to the low expression level of Mnp and Vp in *R. toruloides.* Thus, high-throughput screening is required for fast identification of the engineered strains.

During the batch test, the seed broth was first inoculated into the simulated hydrolysate to reach an initial OD_600_ of 2. However, after fermentation for 96 h, the engineered and the control (NP11) strains were all inhibited by the high concentration of by-products (data were not shown). Due to the additive inhibition caused by inhibitor combinations (Hu and Zhao, 2009), such high concentration of by-products would greatly affect the cell growth in a short time. Moreover, within a certain range, high-density cells could form quorum sensing and improve stress resistance to the environment (Gao et al., 2016). Thus, efforts were made to increase the inoculum amount of fermentation and finally set an initial OD_600_ of 12.

**FIGURE 4:**
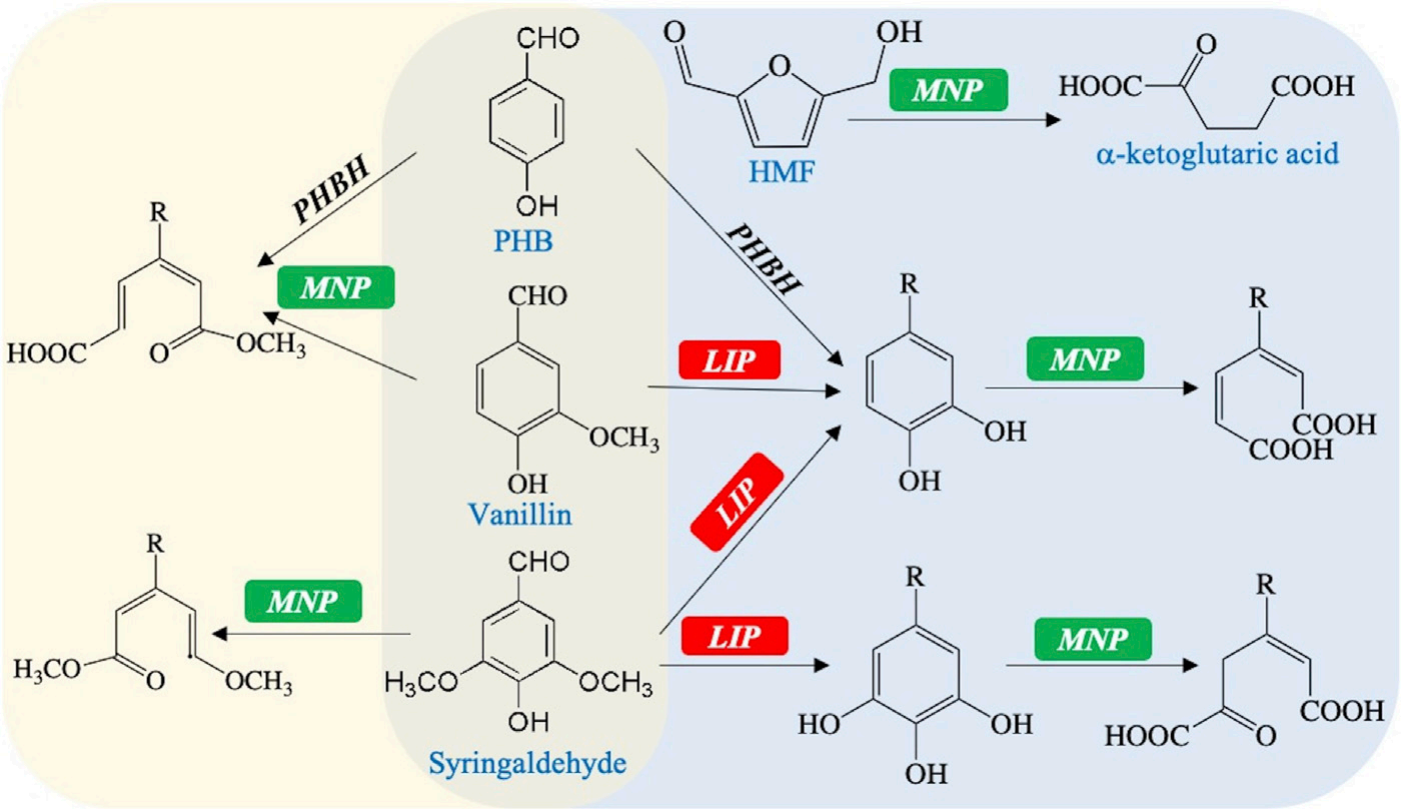
Hypothetical model for the degradation of typical by-products mediated by *MNP* and *VP*: *PHBH*, para-hydroxybenzoate hydroxylase (*R. toruloides* endogenous, gene symbol RHTO_07012); R represents the group of −CHO, −COOH, or −CH_2_OH.

This study also gives a hypothetical model for by-product utilization in the engineered *R. toruloides* (Figure 4). On one hand, benzene rings of phenolic aldehydes would be directly and oxidatively decomposed by Mnp (Supplementary Figure S6A) (Hofrichter, 2002). On the other hand, Lip is capable of demethoxylation or changing the methoxy group into the phenolic hydroxyl group (Supplementary Figure S6B) (Chandra et al., 2017; Wang et al., 2018). Consequently, the hybrid enzyme Vp, which combines the structural–functional properties of Lip and Mnp, could decompose phenolic aldehydes to organic acids. PHB needs to be carboxylated by *PHBH* first and participates in the latter peroxidation ways (Zhu et al., 2012). Otherwise, phenolic aldehydes and alcohols could also be reduced into their less toxic acids by alcohol dehydrogenases, aldehyde reductases, and aldehyde dehydrogenases (Supplementary Figure S6C) (Hu et al., 2018). The HMF might be easily degraded into the available acid by Mnp through its peroxidation effect (Supplementary Figure S6D). These organic acids are able to be further converted into the intermediates to enter the TCA cycle, or into precursor compounds for lipid synthesis. Therefore, the complex by-products of the lignocellulosic hydrolysate would be fast and globally decomposed by the engineered strains with the *VP* gene expression.

In order to further improve the capability of engineered strains, fermentation conditions can be optimized by, for example, medium optimization, pH control, and continuous feeding. Adaptive laboratory evolution (ALE) could also be introduced to promote the robustness of engineered strains (Liu et al., 2021). Through these techniques, oxidases and/or peroxidases, which are major participants in the degradation of lignin-derived phenols such as laccase and dye-decolorizing peroxidases (Dyp), could also be expressed in *R. toruloides*.

## Data Availability

The original contributions presented in the study are included in the article/Supplementary Material; further inquiries can be directed to the corresponding authors.
