# Supplementary material for: Engineering the Oleaginous Yeast Rhodosporidium toruloides for Improved Resistance Against Inhibitors in Biomass Hydrolysates
Source: Front Bioeng Biotechnol. 2021 Nov 15;9:768934. doi: 10.3389/fbioe.2021.768934 (PMC8634367; doi:10.3389/fbioe.2021.768934)
Supplement: Supplementary file 1 [file Table1.DOCX]

Supplementary Tables

**Supplementary Table 1.** Strains and plasmids used in this study.

| Stains | Relevant description | Resource |
| --- | --- | --- |
| *E. coli* DH10B | *F-mcaA Δ (mrr-hsdRMS-mcrBC) Φ80dlacZΔM15 ΔlacX74 recA1 endA1 araD139 Δ (ara, leu)7697 galE15 galK λ- rpsl nupG* | Takara |
| *Rhodosporidium toruloides* NP11 | *MAT A1* | (Zhu *et al.* 2012) |
| *Rhodosporidium toruloides* CGMCC 2.1389 |  | China General Microbiological Culture Collection Centre |
| *Agrobacterium tumefaciens* AGL1 | *AGL0 recA::bla pTiBo542DT Mop + CbR* | (Lin *et al.* 2014) |
| MNP9  MNP28  MNP44  MNP47 | NP11 carrying the  pZPK-MNP-HYG expression cassette | This study |
| VP9  VP28 | NP11 carrying the  pZPK-VP-HYG expression cassette | This study |
| Plasmids |  |  |
| pZPK-MNP-HYG | pZPK-P*_PGK_*-*MNP-**P2A-HYG-*T*_HSP_* | This study |
| pZPK-VP-HYG  pJX14 | pZPK-P*_PGK_*-*VP-P2A-HYG-*T*_HSP_*  pZPK-P*_PGK_*-*HYG-P2A-BLE-*T*_HSP_* | This study  (Jiao *et al.* 2018) |

**Supplementary Table 2.** Primers used in this study.

| Primers | Sequence (5’-3’) | Description |
| --- | --- | --- |
| *P2A*-*HYG*-F  *HYG*-T*_HSP_*-R  P*_PGK_*-*MNP*-F | ctggcgacgtcgaagagaaccctggccctatgccggagctcacggcgac  tgcatgctgcaggtcgactctagaggatcccgcgcacttctctgcactgc  caactcccaccctcccccgtgcagcccaccatgcaccaccatcaccatcac | Amplification of *P2A*-*HYG*-T*_HSP_* for RF clone  Amplification of P*_PGK_*-*MNP*-*P2A* for RF clone |
| *MNP*-*P2A*-R | agggccagggttctcttcgacgtcgccagcctgcttgaggagcgagaagttggtagcgcccgagccggctgggccgttgaactgg |  |
| P*_PGK_*-*VP*-F | caactcccaccctcccccgtgcagcccaccatggccttcgccaagctctc | Amplification of P*_PGK_*-*VP*-*P2A* for RF clone |
| *VP*-*P2A*-R | agggccagggttctcttcgacgtcgccagcctgcttgaggagcgagaagttggtagcgcccgagcccgaaggcgggaccggcggg |  |
| P*_PGK_*-F | ctcgcactcgctctggtacag | Sequencing primers for RF clone |
| *HYG*-R | cgcgcgattcctctccctcc |  |
