# Supplementary material for: Engineering the Oleaginous Yeast Rhodosporidium toruloides for Improved Resistance Against Inhibitors in Biomass Hydrolysates
Source: Front Bioeng Biotechnol. 2021 Nov 15;9:768934. doi: 10.3389/fbioe.2021.768934 (PMC8634367; doi:10.3389/fbioe.2021.768934)
Supplement: Supplementary file 2 [file DataSheet1.pdf]

Supplementary Figures

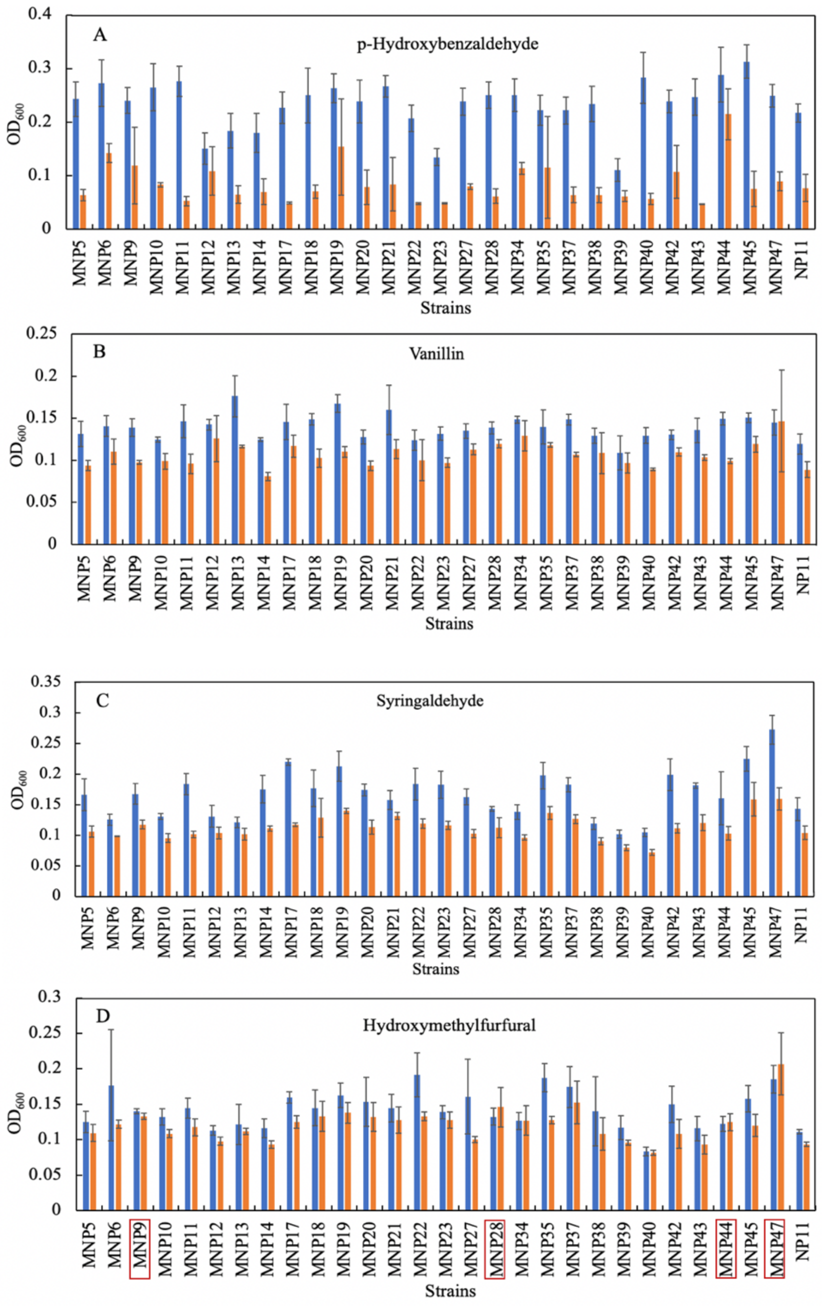

Supplementary Figure 1. Growth of thirty transformants with *MNP* gene integration strains and the control. Yeast were cultured under 2.5 g/L of p-hydroxybenzaldehyde (A), vanillin (B), syringaldehyde (C) and hydroxymethylfurfural (D). Blue columns and orange columns represent initial and final OD_{600}, respectively. Dominant strains were selected and showed by red blocks. Error bars represent the standard deviation of three independent experiments.

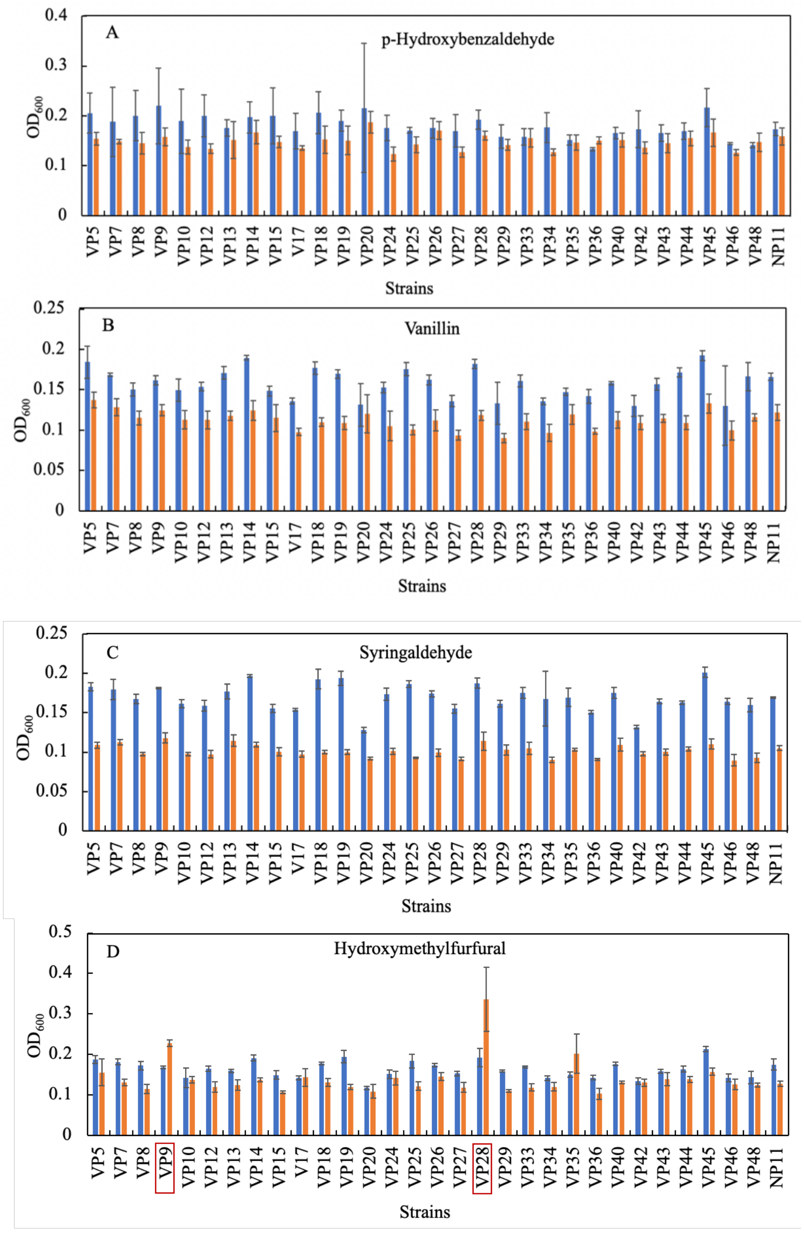

Supplementary Figure 2. Growth of thirty transformants with *VP* gene integration strains and the control. Yeast were cultured under 2.5 g/L of p-hydroxybenzaldehyde (A), vanillin (B), syringaldehyde (C) and hydroxymethylfurfural (D). Blue columns and orange columns represent initial and final OD_{600}, respectively. Dominant strains were selected and showed by red blocks. Error bars represent the standard deviation of three independent experiments.

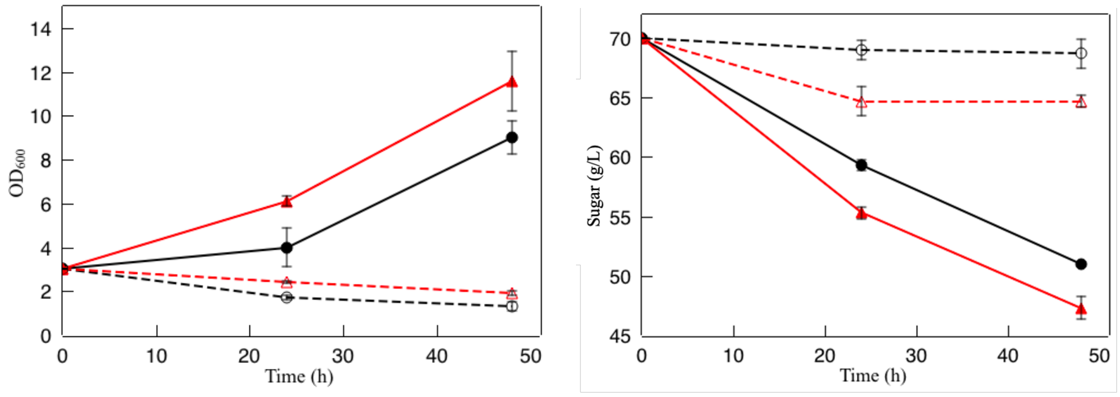

Supplementary Figure 3. Growth of *R. toruloides* NP11(black lines and circles) and CGMCC 2.1389 (red lines and triangles) using concentrated model hydrolysate as culture medium. Various of cell growth (left) and sugar consumption (right). Full lines with solids and dotted lines with hollow spaces represented wild strains under nitrogen-limited medium and concentrated model hydrolysate, respectively. Experiments were done in triple.

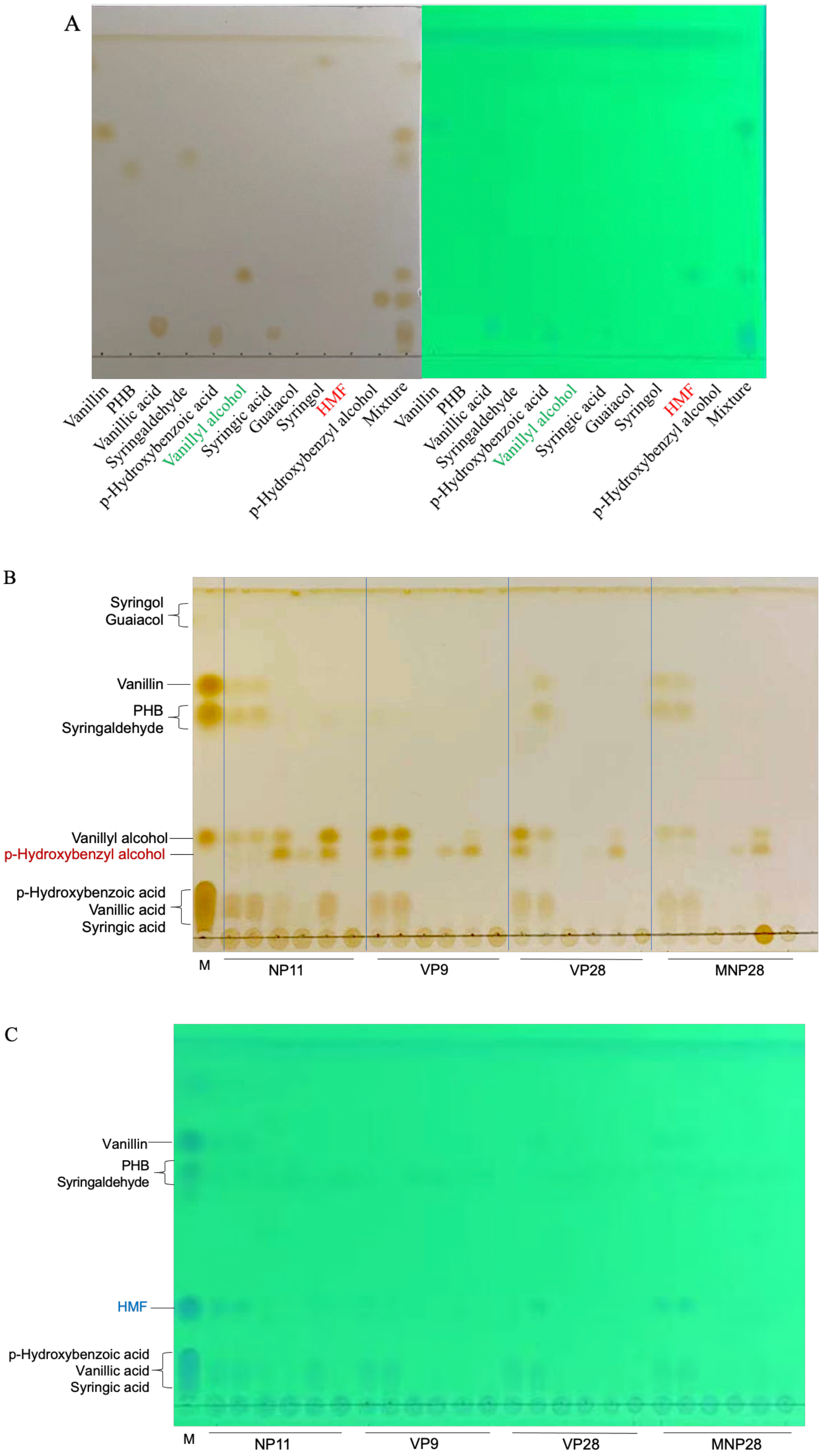

Supplementary Figure 4. TLC analysis of single/mixed standard (A) and by-products variation in fermentation supernatant (B and C). The mixture of by-products was used as marker. Samples were taken at 24-, 48-, 84-, 108-, 132- and 156 h, respectively. The TLC plate was colored by iodine vapour and NDUV under 230 nm. p-Hydroxybenzyl alcohol was newly formed and showed in red. HMF and vanillyl alcohol are on the same position. However, HMF (showed in bule) is visible at 230 nm and vanillyl alcohol is visible after coloring by iodine vapor. They are all degraded in 48 h.

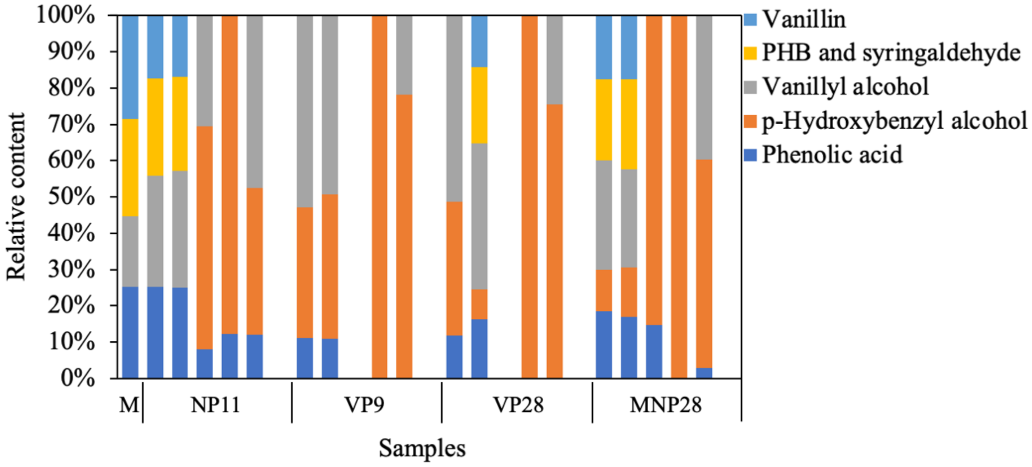

Supplementary Figure 5. Relatively quantitative change of by-products for TLC. Phenolic aldehydes were degraded fastest by Engineered strains VP9 and VP28; Phenolic acids had longest retention time by the controlled strain NP11. Analysis was carried out by ImageJ 1.52—gel grayscale analysis.

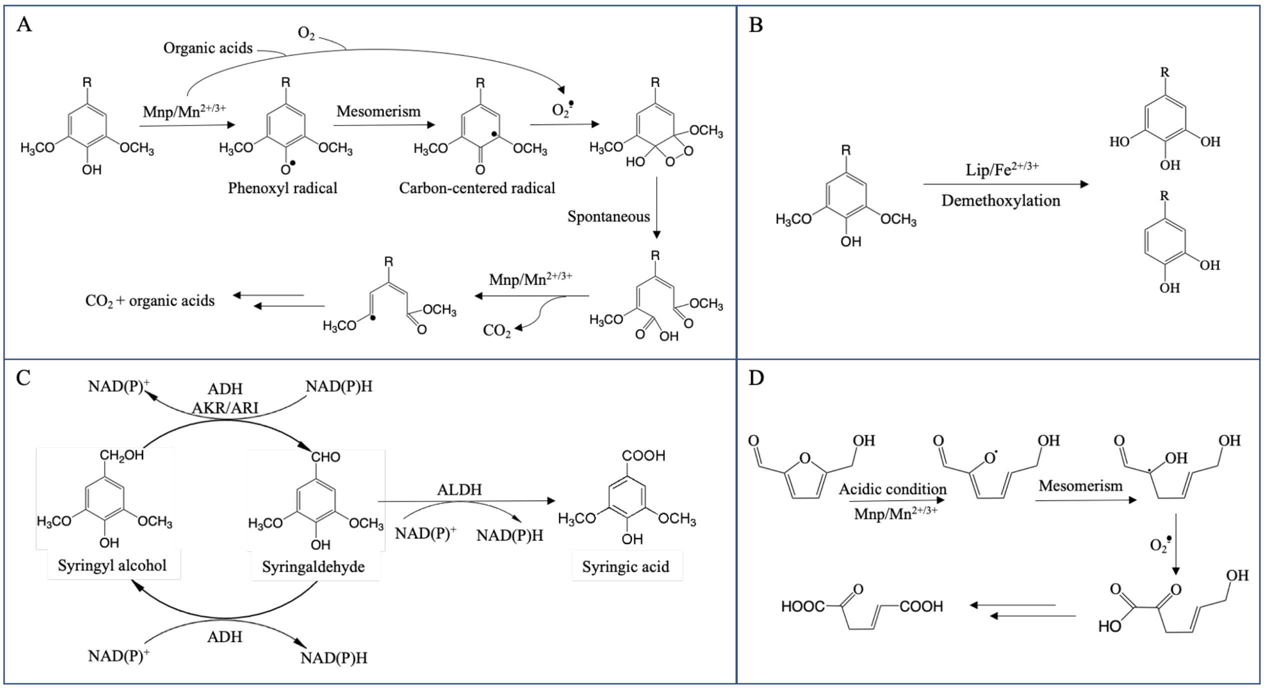

Supplementary Figure 6. Hypothetical model (take syringaldehyde and HMF for examples) for the degradation of typical by-products in engineered strains: A, metabolic mechanism of lignin-derived phenols mediated by *MNP* (Hofrichter, 2002); B, metabolic mechanism of lignin-derived phenols mediated by *LIP* (Wang et al., 2018; Chandra et al., 2017); C, bioconversion of lignin-derived phenolic aldehydes, alcohols and acids mediated by endogenous genes of *R. toruloides* (Hu et al., 2018); D, hypothesis of HMF degradation mediated by *MNP*. R represents the group of -CHO, -COOH or -CH2OH. ADH, alcohol dehydrogenase; AKR, aldehyde dehydrogenase; ARI aldehyde reductase; ALDH, aldehyde dehydrogenase.
